# Plasminogen deficiency reduces disease severity and immune responses in enterovirus A71-infected mice

**DOI:** 10.1128/spectrum.03311-24

**Published:** 2025-05-16

**Authors:** Zheng-Xun Li, Ya-Fang Wang, Li-Ting Chiang, Li-Jin Hsu, Shih-Min Wang, Jen-Ren Wang, Hua-Lin Wu, Shun-Hua Chen, Chuan-Fa Chang

**Affiliations:** 1 Department of Medical Laboratory Science and Biotechnology, College of Medicine, National Cheng Kung University 541413, Tainan City, Taiwan; 2 National Institute of Infectious Diseases and Vaccinology, National Health Research Institutes 50115, Tainan City, Taiwan; 3 Institute of Basic Medical Sciences, College of Medicine, National Cheng Kung University 38026, Tainan City, Taiwan; 4 Department of Pediatric, National Cheng Kung University Hospital, College of Medicine, National Cheng Kung University 34912, Tainan City, Taiwan; 5 Department of Biochemistry and Molecular Biology, College of Medicine, National Cheng Kung University 38026, Tainan City, Taiwan; 6 Department of Microbiology and Immunology, College of Medicine, National Cheng Kung University 38026, Tainan City, Taiwan; 7 University Center for Bioscience and Biotechnology, National Cheng Kung University 34912, Tainan City, Taiwan; Barnard College, Columbia University, New York, New York, USA

**Keywords:** enterovirus A71, plasminogen

## Abstract

Enterovirus A71 (EV-A71) is a causative agent of hand, foot, and mouth diseases. EV-A71 infections may result in severe neurological complications in children. Although several receptors or attachment molecules for EV-A71 have been identified, EV-A71 can still infect host cells even after blocking these receptors with antibodies. We have previously identified plasminogen (PLG), a circulating zymogen of plasmin, as a cell membrane-associated EV-A71-interacting glycoprotein. We confirmed that anti-PLG antibodies could reduce the binding of EV-A71 to RD cells as anti-SCARB2 and anti-nucleolin. Knockdown of PLG reduced EV-A71 binding to RD cells, and preincubation of PLG with EV-A71 increased virus binding. Enzyme-linked immunosorbent assay and surface plasmon resonance assays demonstrated the direct binding of PLG to EV-A71. We further evaluated the biological characteristics of EV-A71-infected PLG knockout (heterozygous) and wild-type mice. We found that the clinical scores and mortality of WT mice were higher than those of PLG-knockout mice after EV-A71 infection. The viral loads in the spinal cord of PLG knockout mice were lower than those in WT mice 6 days post-infection. EV-A71-associated cytokines such as IL-1β, IL-6, MCP-1, IL-10, and IFN-γ were investigated. Serum IL-10 and MCP-1 expression were significantly higher in EV-71-infected WT mice than in PLG knockout mice, and MCP-1 may be one of the critical chemokines that induce intense inflammation and chemoattracts leukocytes. Our findings reveal a possible role for PLG in EV-A71 infection/pathogenesis and shed light on developing novel therapeutic approaches and drugs to prevent EV-A71 infection.

**IMPORTANCE:**

Understanding the pathogenesis of enterovirus A71 (EV-A71) for developing novel drugs or therapeutic approaches has always been a significant issue. In this study, we demonstrated the interactions between plasminogen (PLG) and EV-A71, characterized the biological effects of EV-A71-infected PLG knockout mice, and evaluated their immune response. We found that EV-A71 caused more severe tissue damage than PLG knockout mice in skeletal muscle, spinal cord, and brain stem. Higher virus protein was observed in these tissues of WT mice. The reduced clinical scores, mortality, and cytokine expression suggested PLG may be involved in EV-A71 infection-induced cytokine storm. The findings and animal model in the current study provide the new drug target for anti-EV-A71 drug discovery.

## INTRODUCTION

Enterovirus 71 (EV-A71), an RNA virus of the *Picornaviridae* family, causes hand-foot-and-mouth disease (HFMD) (1). Occasionally, infection can lead to severe complications, including encephalitis, aseptic meningitis, pulmonary edema or hemorrhage, and acute flaccid paralysis (2, 3). Outbreaks of EV-A71 infections have occurred worldwide since 1997 (4–7). Several large epidemics of EV-A71 have been reported in the Asia-Pacific region, including Malaysia (8), Vietnam (9), and Taiwan (10, 11). Since 1998, EV-A71 has become a public health threat in children each year, and most patients are younger than 5 years old (10, 12–15).

In 2009, Shimizu and colleagues found that human P-selectin glycoprotein ligand-1 (PSGL-1) is a functional receptor of EV-A71 (16). They also found that tyrosine sulfate on the terminal residue of PSGL-1 was critical for EV-A71 infection (17). Koike et al. found that scavenger receptor class B2 (SCARB2) is a cellular receptor of EV-A71 (18). In addition, several adhesion molecules that mediate the attachment of EV-A71 to host cells have been reported, including sialylated glycoprotein, heparin sulfate, and annexin A2 (ANXA2) (19–21). However, treating cells with anti-PSGL-1, anti-SCARB2, and anti-ANXA2 monoclonal antibodies or lectins did not entirely block the infection in EV-A71 host cells. These phenomena imply that some undiscovered receptors or cofactors participate in the attachment and infection of EV-A71 (22).

Our previous study demonstrated that cell surface sialic acids could mediate EV-A71 binding and infection (19). Using targeted glycoproteomic approaches, we identified several EV-A71 interacting glycoproteins, including nucleolin (NCL), selenoprotein S (SELS), and plasminogen (PLG) (23). PLG is a single-chain glycoprotein of 810 amino acids; the mature form of PLG, plasmin, contains 791 amino acids due to the cleavage of a leader peptide during secretion (24). It is synthesized in the liver (25) and at a high blood concentration (2.4 µM). PLG is broadly expressed in extrahepatic tissues, such as the spleen, kidney, and brain (26). PLG and plasmin play roles in fibrinolysis and hemostasis, cell migration via degradation of the extracellular matrix, tissue remodeling, wound healing, angiogenesis, macrophage recruitment during inflammation (27, 28), tumor cell invasion and metastasis (29), neurite outgrowth, and skeletal myogenesis (30, 31).

Although PLG is not a primary viral receptor that directly interacts with viral proteins, several studies have shown that PLG is a cofactor that facilitates virus binding or infection. For instance, plasmin, the active form of plasminogen, can cleave hemagglutinin into its active form, enabling influenza viral entry (32, 33). The viral glycoproteins of herpes simplex virus (HSV) can bind PLG or host cell-associated PLG-binding proteins and activate PLG to plasmin, which degrades extracellular matrix and facilitates HSV infection. Antibodies produced during dengue virus (DENV) infection may cross-react with PLG, enhancing its activation to plasmin, increasing vascular permeability, and contributing to the hemorrhagic manifestations of severe dengue (34). In addition, activating PLG to plasmin in SARS-CoV-2 infection promotes coagulopathy, inflammation, and tissue damage, contributing to severe COVID-19 outcomes. This interaction highlights PLG’s role as a mediator in viral pathogenesis and a potential target for therapeutic intervention.

We previously identified PLG as an EV-A71 interacting protein using glycoproteomic approaches. The current study demonstrated that EV-A71 directly interacts with PLG based on immunoprecipitation, enzyme-linked immunosorbent assay (ELISA), and surface plasmon resonance (SPR) results. Anti-PLG antibodies can abolish the binding of EV-A71 to host cells. PLG knockdown reduced the binding of EV-A71 to RD cells, and preincubation of PLG with EV-A71 increased virus binding to host cells. To dissect the roles of PLG in the immune responses of EV-A71 infection, the clinical scores, mortality, tissue viral loads, and cytokine expressions of EV-A71 infected PLG knockout (PLG-KO) and wild-type (WT) mice were also evaluated.

## MATERIALS AND METHODS

### Cell culture, virus amplification, and purification

Human muscle rhabdomyosarcoma (RD; ATCC, No. CCL-136) cells were grown and maintained in DMEM with a low level (2.2 g/L) of sodium bicarbonate at pH 7.4, 10% fetal bovine serum (FBS), and 1% penicillin-streptomycin. Infectious clones MP4 (mouse-adapted strain) and 6356 (human strain, C2 genotype) were propagated in RD cells, grown in viral growth medium (DMEM with 1% penicillin-streptomycin and 2% FBS), and incubated at 35°C until over 90% of cytopathic effects formation. The viral supernatants were collected after centrifugation at 8,000 rpm for 20 min. Viral titers were determined using a plaque-forming assay.

### ELISA

The 96-well plates (Nunc-Immuno) were coated with 50 µL/well of purified virus and BSA with coating buffer (0.1 M NaH_2_PO_4_/Na_2_HPO_4_, pH = 9.0) overnight at 4°C. The supernatants were removed, and the wells were washed three times with 200 µL/well wash buffer (0.05% Tween20 in PBS, pH = 7.0). The plates were incubated with 100 µL/well of blocking buffer (3% BSA in PBS, pH = 7.0) for 2 h at 37°C. The supernatants were removed, and the wells were washed three times. Human PLG proteins (100 µL/well, P4958, Abnova) were added to each well, and the plates were incubated overnight at 4°C. After washing the unbound proteins three times, the plates were incubated with anti-PLG antibody (ab77811, Abcam) for 1 h at 37°C. The wells were rinsed three times and incubated with HRP-conjugated anti-mouse IgG1 antibody (GTX35185, GeneTex) for 45 min at 37°C. The wells were washed and incubated with 50 µL/well tetramethyl benzidine (TMB, Sigma). The reaction was stopped by adding 50 µL/well of 1 M H_3_SO_4_, and the optical density at 450 nm was measured using an ELISA reader.

### Plaque assay and 50% cell culture infective dose (CCID_50_)

RD cells (1 × 10^5^ cells/well, 1 mL) were seeded in 12-well plates, incubated at 37°C for 16–18 h, and 800 µL of culture medium was removed. One hundred microliters of each 10-fold serially diluted sample were added to 2% FBS DMEM in the wells. After absorption for 1 h at 37°C, the medium was removed, and the cells were washed with serum-free medium. An Overlay medium containing 2% FBS and 1.5% methylcellulose was added and incubated at 37°C for 72 h. The overlay medium was discarded and fixed with a solution containing 10% formaldehyde (1 mL/well) at room temperature for 6–8 h and then discarded and stained with 1% crystal violet in methanol at room temperature for 6–8 h. The plates were washed with flowing water and dried to count the plaques (PFU/mL).

### SPR assay

Microtitration plates (384 wells) were coated with 1 ng/well of PLG and then incubated with twofold serially diluted EV-A71 viral particles. The interactions were detected using an SPR reader (EnSpire or BIAcore). SPR signals were expressed in response units (RU).

### Knockdown of PLG

For shRNA transfection, RD cells (2.2 × 10^5^ cells/well) were seeded in a six-well plate. pLKO.1-puro-shPLG plasmids pre-mixed with TurboFect transfection reagent (Thermo Fisher Scientific) for 20 min were added to each well and cultured for 48 h at 37°C. Lentivirus-infected RD cells (2 × 10^5^ cells/well) were seeded in a six-well plate. The 100 µL of lentivirus-based shPLG and 1.6 µL of polybrene were added to each well containing 2 mL 10% FBS-DMEM. After 24 h, the cells were selected in the presence of DMEM supplemented with 2 µg/ml puromycin (Sigma) (Fig. S1).

### Western blotting

Protein lysates were resuspended in 6× sample buffer, denatured for 5 min at 95°C, and separated by 8–12% SDS-PAGE using electrophoresis. Proteins were transferred to PVDF membranes (Millipore) for 90 min, and the membranes were blocked for 1 h at room temperature in 5% milk. Primary antibodies against VP2 of EV-A71 (MAB979, Millipore), VP1 of EV-A71 (bs-2297r, Bioss), and PLG (ab77811, Abcam) were incubated with the membranes overnight at 4°C. The membranes were washed three times and incubated with HRP-conjugated secondary antibodies for 1 h at room temperature. The membranes were washed and analyzed using a LAS-3000 chemiluminescence system (FUJIFILM Life Science).

### Animals

PLG-deficient mice (homozygous, P0) were provided by Dr. Hua-Lin Wu, who purchased them from The Jackson Laboratory (Bar Harbor, ME, USA). We obtained PLG-KO (heterozygous, P1) mice by mating PLG-deficient mice with WT mice. We mated PLG-KO mice with PLG-KO mice (P1 × P1) to obtain P2 mice that contained WT, PLG-KO mice (heterozygous), and PLG-deficient mice (homozygous) at the same birth. P2 mice were used for EV-A71 infection throughout the experiments (Fig. S2). The EV71 mouse-adapted MP4 strain (5 × 10^4^ pfu/50 µL/mouse) was used to infect 7-day-old PLG-KO and WT mice via intraperitoneal (i.p.) injection. The clinical scores, body weights, and survival rates were recorded.

### Genotyping

Seven-day-old WT and PLG-KO mice were genotyped by polymerase chain reaction (PCR) using the standard genotyping protocol of The Jackson Laboratory. Genomic DNA was isolated from the tail of each mouse. Mouse tails were dissolved in 40 µL protein kinase K with PCR Buffer with Nonionic Detergents (PBND) and incubated at 95°C for 16 h. The samples were centrifuged at 14,000 rpm for 10 min. The supernatants were collected for genotyping by PCR (Table S1). WT mice express a single band on 268 bp, PLG-KO mice express a single band on 190 bp, and hetero mice will express two bands on 190 and 268 bp.

### Viral load in tissues

Seven-day-old PLG-KO and WT mice were infected with 5 × 10^4^ pfu/50 µL/mouse EV-A71 via i.p. injection. The mice’s brain stem, spinal cord, and skeletal muscle were collected in 1 mL of 2% FBS-DMEM using a tissue grinder after sacrifice at 2, 4, and 6 days post-infection. Freezing and thawing of the tissue sample were repeated three times, and the viral supernatants were collected after centrifugation at 4,000 rpm for 30 min at 4°C to remove tissue debris. Viral titers were determined using plaque assays.

### Histopathological and immunohistochemical (IHC) staining

The mice were euthanized using CO_2_ at 2, 4, and 6 days post-infection. The brain stem, spinal cord, and skeletal muscle were collected and fixed in 10% formaldehyde. Tissue immunohistochemistry (IHC) was performed on 4 μm thick formalin-fixed paraffin-embedded sections. The primary antibodies were monoclonal mouse anti-human EV-A71 (MAB979, Millipore) and rabbit anti-human PLG polyclonal antibodies (GTX102877, GeneTex). The slides were stained with hematoxylin and eosin (H&E) or IHC in a tissue bank at NCKU. Slides were incubated with primary antibodies at room temperature for 8 min, followed by hydroperoxide blocking for 5 min, and developed with 3,3′-diaminobenzidine chromogen (D5905, Sigma-Aldrich) for 10 min using the Bond Polymer Refine Detection Kit. Counterstaining was performed using hematoxylin.

### Cytokine measurement

Seven-day-old PLG-KO and WT mice were infected with 5 × 10^4^ pfu/50 µL/mouse EV-A71 strain via i.p. injection. The mice were sacrificed 2, 4, and 6 days post-inoculation. Cardiac puncture was used to collect serum from mice. After clot formation, serum samples were collected by centrifugation at 2,000 × *g* for 10 min. After thawing three times, the spinal cord, brain stem, and skeletal muscle were collected in Tissue Protein Extraction Reagent (T-PER) (Thermo Fisher Scientific). The tissues were ground using a tissue grinder and homogenized at 25 Hz for 0.5 min. The spinal cord and skeletal muscle homogenates were centrifuged at 4,000 rpm for 30 min at 4°C to obtain supernatants. The supernatants and serum samples were assessed using SimpleStep ELISA kits (cytokine ELISA kit MCP-1, IL-1β, IL-6 Abcam) and fully validated and ready-to-use ELISA kits (cytokine ELISA kit IL-10, IFNG). Briefly, antibody-specific mouse cytokines were added to a 96-well plate. The captured antibody was incubated with the standards and samples for 2.5 h at room temperature or overnight at 4°C. The wells were washed, and biotinylated anti-mouse cytokine antibody was added. After washing away the unbound biotinylated antibodies, HRP-conjugated streptavidin was pipetted into the wells. The wells were rewashed, and TMB substrate solution was added. The developed color was proportional to the number of bound cytokines. The stop solution changed color from blue to yellow, and the intensity of the color was measured at 450 nm. The concentrations of these cytokines in the samples were calculated from standard curves of known concentrations.

### Statistical analysis

The Student’s *t*-test was used to analyze the differences between the two treatment groups using GraphPad Prism 5 software. Statistical significance was set at 0.05. All the assay results were obtained from at least three independent experiments.

## RESULTS

### Anti-PLG antibody reduces EV-A71 binding to RD cells

**Fig 1:**
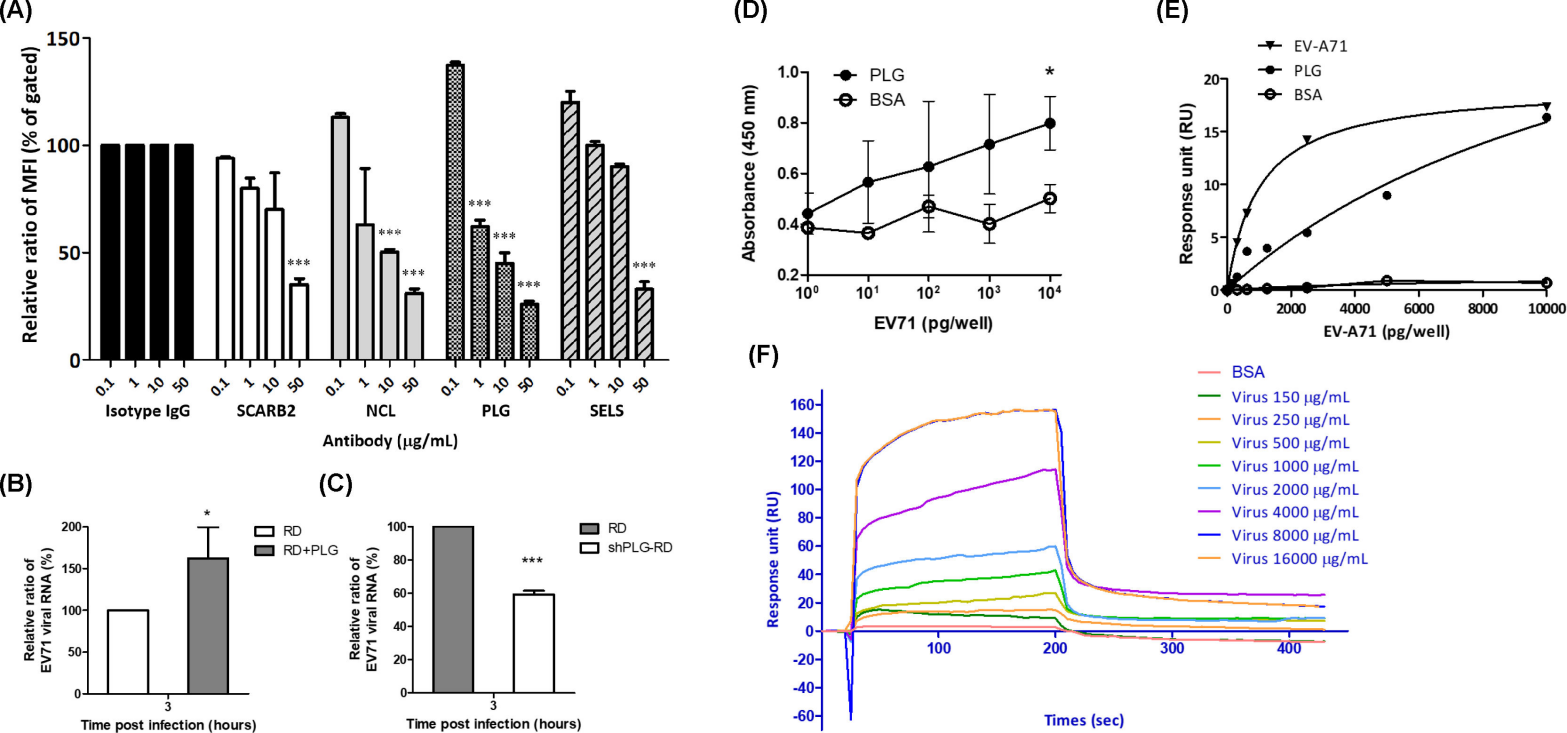
Anti-plasminogen antibody blocks the binding of EV-A71 to RD cells. (**A**) RD cells were treated with different concentrations of antibodies, including anti-SACRB2, anti-NCL, anti-PLG, and anti-SELS antibodies, for 1 h, followed by infection with EV-A71 for 3 h at 4°C. The bound virus was detected by flow cytometry with anti-EV-A71 antibody and FITC-conjugated second antibody. Preincubation of antibodies significantly reduced the binding of EV-A71 to RD cells in a dose-dependent manner. (**B**) PLG-virus preincubation facilitates EV-A71 binding to RD cells. (**C**) Knockdown of PLG attenuates EV-A71 binding to RD cells. (**D**) Virus-protein interaction detected by ELISA assay. Purified EV-A71 and BSA were diluted from 1 pg/50 µL to 10 ng/50 µL. The 100 pg/100 µL of PLG was added to each well. EV-A71 directly interacts with PLG. (**E**) The plates were coated with 1 ng/well of PLG. The purified EV-A71 was diluted in twofold serial dilution. The virus-protein interaction was detected by an SPR assay reader (EnSpire). (**F**) The virus-protein interaction was detected by the SPR assay reader (BIACore). The data were expressed as the mean ± SD of each group (*n* ≥ 3). *P < 0.05.

To evaluate whether the anti-PLG antibody influences the attachment of EV-A71 to host cells, RD cells were treated with or without specific antibodies, including anti-SCARB2 (B97306, Sigma), anti-NCL (ab22758, Abcam), anti-PLG (ab77811, Abcam), and anti-SELS (SAB2102105, Merck) antibodies for 1 h at 4^o^C, and then incubated with EV-A71 for 3 h at 4^o^C. The bound virus was detected by flow cytometry using an anti-EV-A71 antibody (MAB979, Millipore) and FITC-conjugated second antibody (C04025, Croyez). Preincubation with the anti-PLG antibody significantly reduced the binding of EV-A71 to RD cells in a dose-dependent manner (Fig. 1A). In addition, anti-SCARB2, anti-NCL, and anti-SELS antibodies inhibited EV-A71 binding.

### Preincubation of EV-A71 with PLG increases the binding of virus to host cells

To evaluate whether PLG facilitates EV-A71 infection, EV-A71 6356 was preincubated with PLG (200 µg/mL, 2.4 µM) in serum-free DMEM at 37^o^C for 1 h. The mixture was incubated with RD cells at 4°C for 3 h, and the unbound virus was removed with PBS. The viral RNA was determined by real-time PCR. We found that the viral RNA in the PLG preincubated group was significantly higher than that of the PLG-untreated group at 3 h post-infection (Fig. 1B). We further determined EV-A71-induced cytopathic effects (CPEs) with or without PLG preincubation. However, the CPE observed in the PLG-preincubated group was the same as in the PLG-untreated group at 24 h post-infection (Fig. S3A). The viral RNA of the PLG preincubated group showed no significant difference from that of the PLG-untreated group at 0, 24, and 36 h post-infection (Fig. S3B). This result indicates that additional PLG may be involved in the binding of EV-A71 to RD cells but not subsequent infection.

### Knockdown of PLG reduces EV-A71 attachment to RD cells

To establish PLG knockdown cell lines, RD cells were transfected with PLG shRNA. The transfection efficiency was determined using a GFP-based plasmid (Fig. S1A). PLG expression was reduced by 32% in RD cells when 6 µg of PLG shRNA plasmid was transfected (Fig. S1B). We also applied a lentivirus-based shRNA strategy to obtain a stable PLG-knockdown cell line. RD cells were infected with lentivirus expressing PLG shRNA, and the stable clone was selected using puromycin treatment. PLG expression of PLG was steadily reduced by 35% in RD cells (Fig. S1C). To evaluate the effects of PLG knockdown on EV-A71 binding, shPLG-RD cells were incubated with EV-A71 6356 at 4°C for 3 h, and the viral RNA was evaluated using RT-PCR. The viral RNA of shPLG-RD cells was significantly lower than that of RD cells (Fig. 1C). Therefore, PLG knockdown in RD cells should be involved in EV-A71 binding to host cells.

### EV-A71 directly interacts with PLG

To reduce the influence of bovine PLG (from FBS), EV-A71 amplified in serum-free DMEM was used in the following assays. The interaction between EV-A71 and PLG was dose-dependent according to the ELISA assay (Fig. 1D). BSA was used as a negative control for PLG. The PLG-EV-A71 viral particle interaction also showed a dose-dependent effect, as measured by the EnSpire system (Fig. 1E). In addition, the association rate constant (*K*_on_), dissociation rate constant (*K*_off_), and dissociation constant (*K*_D_) of EV-A71 6356 bound with PLG measured by BIACore systems were 1.73 × 10^6^ (M^−1^ min^−1^), 3.38 × 10^−3^ (min^−1^), and 1.96 × 10^−9^ (M), respectively (Fig. 1F). Interestingly, coxsackievirus A10 and A16 also interacted with PLG, measured using the EnSpire system (Fig. S4) (35). These results suggest that EV-A71 directly interacts with PLG.

### PLG-KO increases the survival rates in EV-A71-infected mice

**Fig 2:**
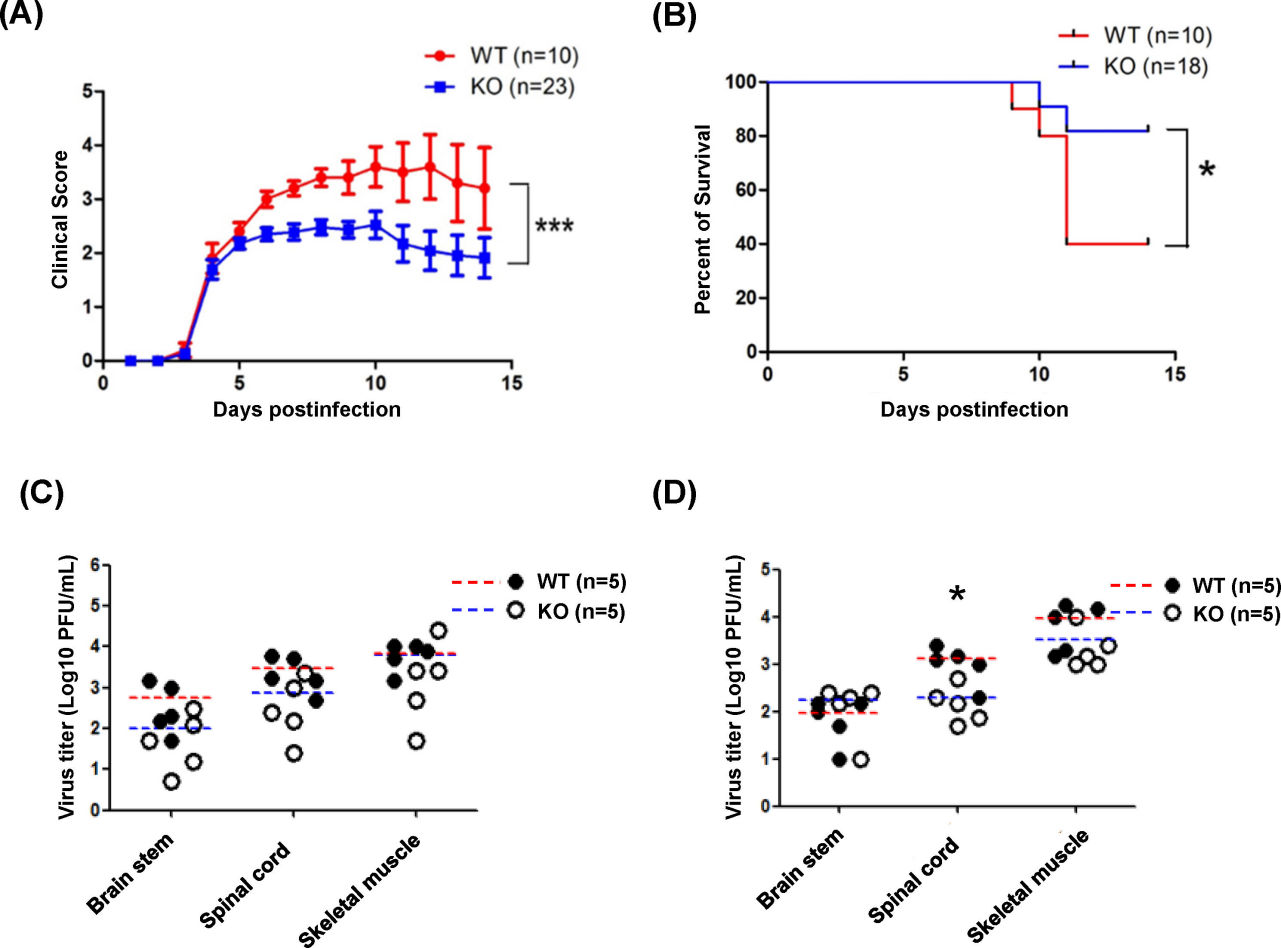
The clinical scores, survival rates, and tissue viral loads of PLG-KO and WT mice in EV-A71 infection. Seven-day-old WT and PLG-KO mice were infected with the 5 × 10^4^ pfu/mouse of EV71 strain. The clinical score (**A**) and the survival rates (**B**) of the WT and PLG-KO mice were measured for 2 weeks. PLG-KO mice showed lower clinical scores and mortality rates than WT mice. Clinical scores: 0, healthy; 1, reduced motility; 2, limb weakness; 3, limb paralysis; 4, moribund; and 5, death. Seven-day-old WT and PLG-KO mice were infected with 5 × 10^4^ pfu/mouse of EV-A71 MP4 strain to evaluate the viral loads in the brain stem, spinal cord, and skeletal muscle. The WT (*n* = 5) and PLG-KO mice (*n* = 5) were sacrificed 3 days (**C**) and 6 days (**D**) post-infection. The virus titers were determined by plaque assay. The mean of virus titers (log10 pfu/g) in WT mice was red, and in PLG-KO mice was blue. *P < 0.05, **P < 0.01, and ***P < 0.001.

To further explore the effects of PLG in EV-A71 infection *in vivo*, we mated PLG-KO mice with PLG-KO mice to obtain WT, PLG-KO (heterozygous), and PLG deficiency mice at the same birth (Fig. S2). The body weights of the WT and PLG-TG mice were recorded for 5 weeks. Like WT mice, PLG-KO mice showed normal body weight and appearance (Fig. S5). To explore the optimal condition of EV-A71 infection, we evaluated the clinical scores of 7-day-old WT mice inoculated with different viral loads of EV-A71 (from 2.5 × 10^2^ to 2.5 × 10^6^ pfu/mouse). The optimal condition was observed in mice infected with 5 × 10^4^ pfu/mouse of EV-A71 MP4. All the mice died within 5 days post-infection if the viral titers were higher than 5 × 10^4^ pfu/mouse (Fig. S6). Seven-day-old WT and PLG-KO mice were infected with EV-A71 (5 × 10^4^ pfu/mouse) intraperitoneally, and the clinical scores and survival rates were monitored for two weeks. We found that the clinical scores of WT mice were higher than those of PLG-KO mice and significantly elevated 6–8 days post-infection (Fig. 2A). The mortality of EV-A71-infected PLG-KO mice (20%) was much lower than EV-A71-infected WT mice, in which 60% died within 11 days post-infection (Fig. 2B).

### Virus titers in organs of EV-A71-infected PLG KO and WT mice

To investigate the viral load in WT and PLG KO mice after EV-A71 infection, the mice were sacrificed on days 3 and 6 post-infection. The average levels of virus titer in the skeletal muscle, spinal cord, and brain stem showed no significant difference between EV-A71-infected WT and PLG KO mice 3 days post-infection (Fig. 2C). The virus titers in skeletal muscle and brain stem also exhibited no difference between WT and PLG KO mice 6 days post-infection (Fig. 2D). Notably, the virus titers in the spinal cord of WT mice were significantly higher than PLG KO mice 6 days post-infection (Fig. 2D).

### Disease progression in organs of EV-A71-infected WT and PLG-KO mice

**Fig 3:**
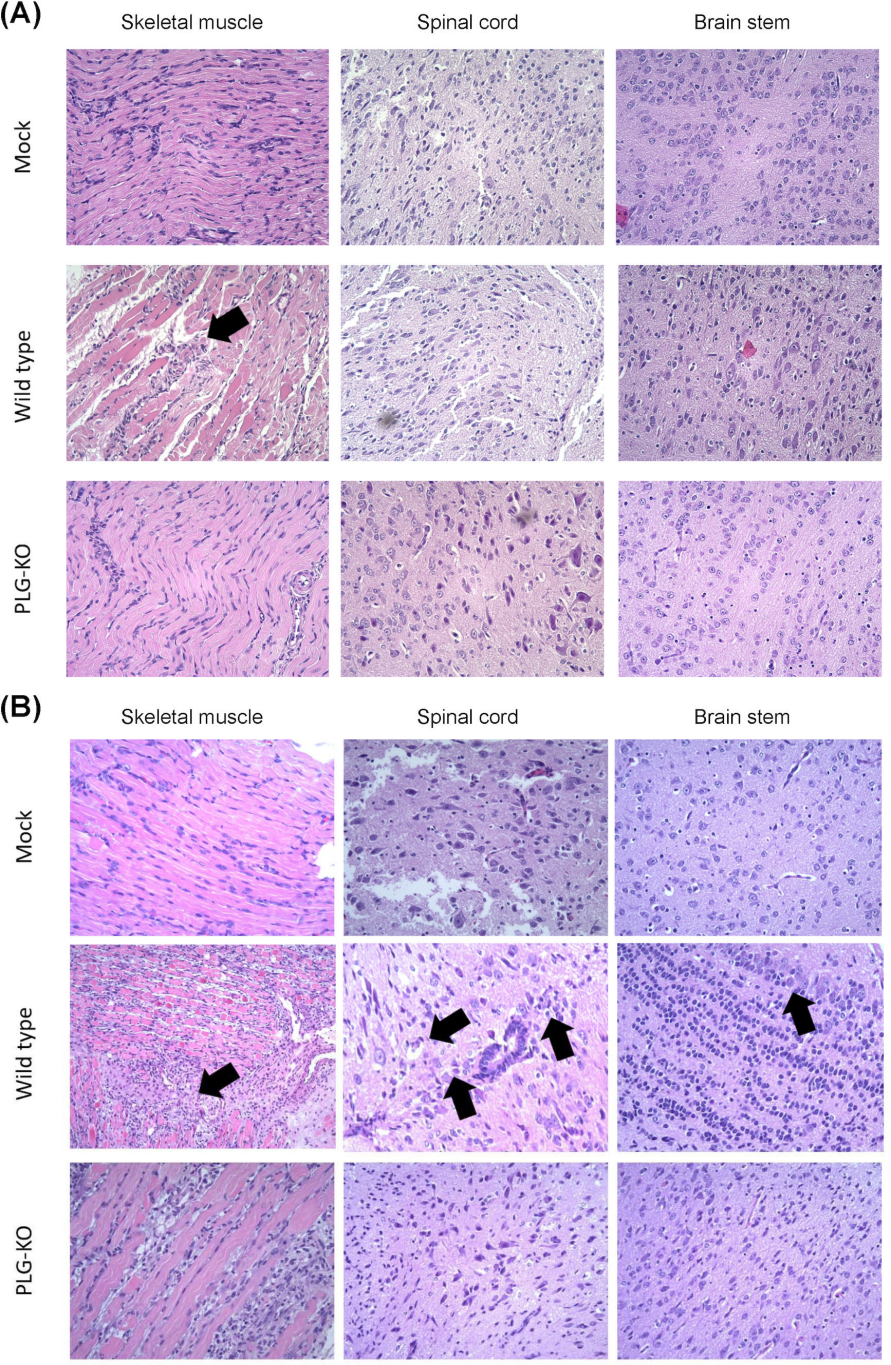
PLG-KO decreases the disease progression of EV-A71-associated neuropathy. Seven-day-old WT and PLG-KO mice were infected with 5 × 10^4^ pfu/mouse EV-A71 intraperitoneally. Mice were sacrificed on days 2 (**A**) and 6 (**B**). Paraffin-embedded skeletal muscle, spinal cord, and brain stem sections were examined with H&E stain at 200× magnifications. WT mice show cell neuropathy, such as proliferation, tissue damage, and lymphocytic infiltration in their nervous system at day 6 post-infection (arrow indicated). Negative control (mock): without virus infection.

Disease progression in mock, EV-A71-infected PLG-KO, and WT mice was investigated. The mice were sacrificed on days 2 (Fig. 3A) and 6 (Fig. 3B) post-infection. The skeletal muscle, spinal cord, and brain stem were collected and processed with formalin fixation, paraffin embedding, H&E, and IHC staining. On day 2 post-infection, the muscle fibers of the mock were intact, and the tissue appeared well-organized and had normal architecture (Fig. 3A, top left). Increased inflammation with noticeable immune cell infiltration, disrupted muscle fiber architecture, and tissue damage were observed in EV-A71-infected WT mice (Fig. 3A, middle left). In EV-A71-infected PLG-KO mice, the muscle tissue showed well-preserved architecture without inflammation or damage compared to WT mice (Fig. 3A, bottom left). The WT mice showed more muscle damage and inflammation following EV-A71 infection than the mock and the PLG-KO mice, which may lead to differences in disease outcome.

In the spinal cord section, the tissue showed normal morphology with intact neurons and glial cells for mock (Fig. 3A, top middle). No visible signs of inflammation, degeneration, or neuronal loss were observed. Immune cell infiltration-induced pathological changes, including neuronal degeneration, vacuolization, and increased cellularity, were observed in the spinal cord section of EV-A71-infected WT mice (Fig. 3A, center). The dark spot found in the image may indicate necrotic tissue or cellular debris caused by virus infection. Compared to EV-A71-infected WT mice, the spinal cord tissue in PLG-KO mice showed relatively preserved morphology, similar to the mock (Fig. 3A, bottom middle). The same phenomenon was observed in the brain stem sections of the mock, EV-A71-infected WT, and PLG-KO mice (Fig. 3A, right). The brain stem tissue appears normal, with well-preserved neurons and intact structures in mock and EV-A71-infected PLG-KO mice (Fig. 3A, top right and bottom right). However, neuron loss and vacuolization were observed in the brain stem section of EV-A71-infected WT mice (Fig. 3A, middle right). A prominent dark red structure was visible, suggesting hemorrhage, necrotic tissue, or immune cell infiltration in response to EV-A71 infection. The skeletal muscle, spinal cord, and brain stem of EV-A71-infected WT mice represented significant tissue damage and inflammation compared to mock and EV-A71-infected PLG-KO mice under the same clinical scores.

On day 6 post-infection, the skeletal muscle, spinal cord, and brain stem sections of the mock showed well-organized tissue structure and intact cell morphology without immune cell infiltration (Fig. 3B, top left, top middle, and top right). Compared to day 2, the muscle tissue of EV-A71-infected WT mice exhibited more significant pathological changes induced by inflammation, such as disrupted muscle fiber architecture, immune cell infiltration, and tissue necrosis/degeneration on day 6 post-infection (Fig. 3B, middle left). We also observed the loss of normal neuronal architecture, the increased presence of inflammatory cells surrounding neurons, and the disrupted structures in the spinal cord section of EV-A71-infected WT mice (Fig. 3B, center). The accumulation of inflammatory cells, neuronal damage, and increased cellularity in the brain stem section of WT mice may indicate severe inflammation, cellular infiltration, and neuronal degeneration induced by EV-A71 infection (Fig. 3B, middle right). These observations corresponded with the findings in viral load determination (Fig. 2D). Although the clinical score was elevated, the muscle tissue of EV-A71-infected PLG-KO mice appeared relatively less affected than WT mice (Fig. 3B, bottom left). The neurons and surrounding tissue in the brain stem showed more intact and lower immune cell infiltration, indicating minor inflammation in EV-A71-infected PLG-KO mice than in WT mice (Fig. 3B, bottom middle).

**Fig 4:**
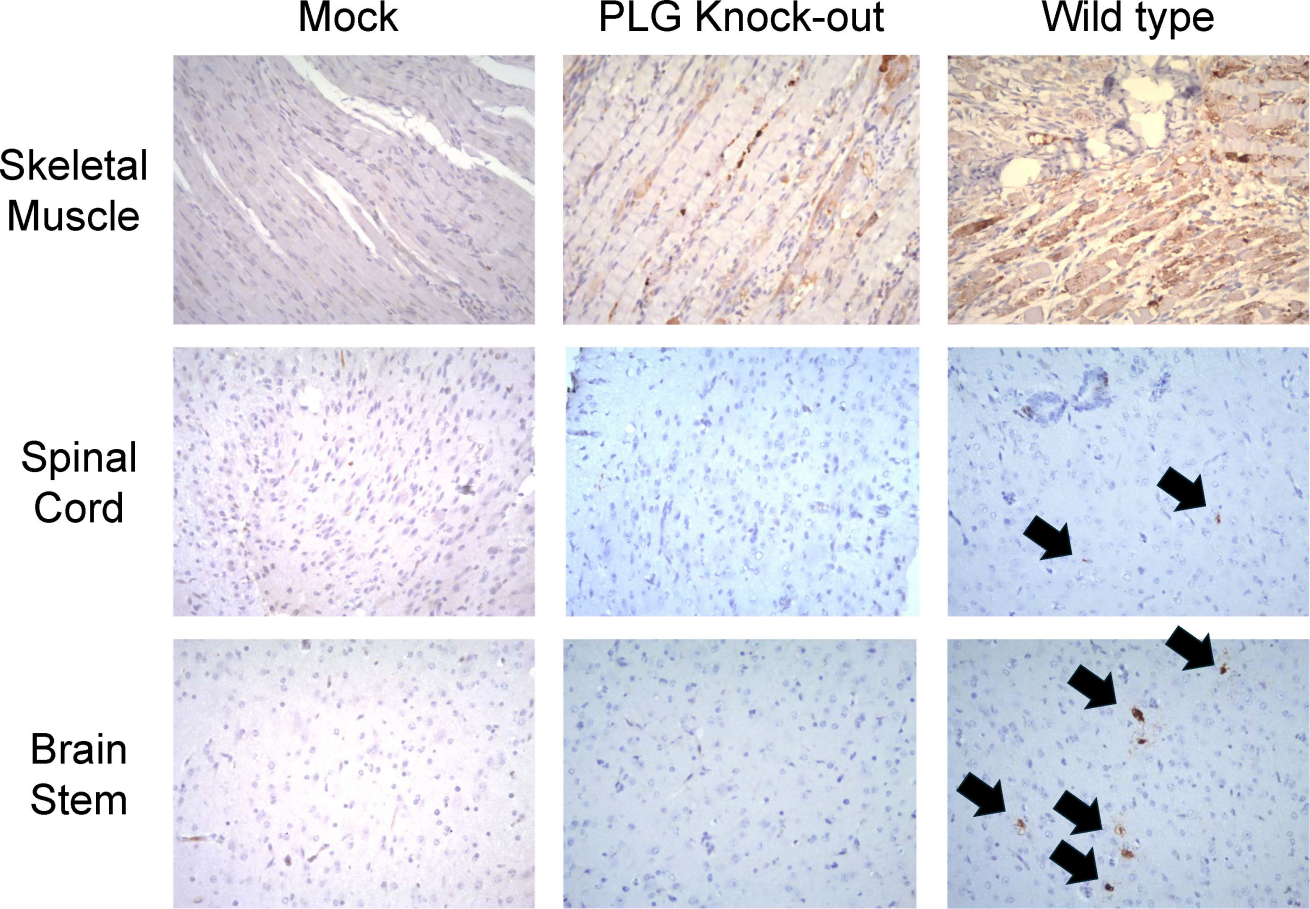
PLG-KO decreases EV-A71 VP1 protein expression in tissues. Seven-day-old WT and PLG-KO mice were infected with 5 × 10^4^ pfu/mouse EV-A71 intraperitoneally. Mice were sacrificed on day 6. Paraffin-embedded skeletal muscle, spinal cord, and brain stem sections were examined with IHC stain at 200× magnifications. IHC staining detected the most vital expression of viral VP1 protein in skeletal muscle, spinal cord, and brain stem in the WT mice (arrow indicated). Negative control (mock): without virus infection.

We further analyzed the viral protein VP1 in the organ sections of mock, PLG-KO, and WT mice. Minimal to no staining was observed in skeletal muscle tissue of mock (Fig. 4, top left). Moderate staining was observed, and the muscle fibers appeared relatively preserved in skeletal muscle tissue of PLG-KO mice (Fig. 4, top middle). Intense staining was observed, which indicated severe EV-A71 infection in the skeletal muscle tissue of WT mice (Fig. 4, top right). In the spinal cord section of mock and PLG-KO mice, minimal to no detectable staining was observed, suggesting a protective effect of PLG-KO against EV-A71 infection (Fig. 4, middle left and center). Positive staining could be found on the spinal cord section of WT mice, suggesting the presence of EV-A71 VP1 protein (Fig. 4, middle right). Like the IHC staining in the spinal cord, none of the signal could be detected in the brain stem sections of mock and PLG-KO mice (Fig. 4, bottom left and bottom middle). However, higher VP1 signals were observed in the brain stem sections of EV-A71-infected WT mice (Fig. 4, bottom right).

### Immunologic response of EV-A71-infected WT and PLG-KO mice

**Fig 5:**
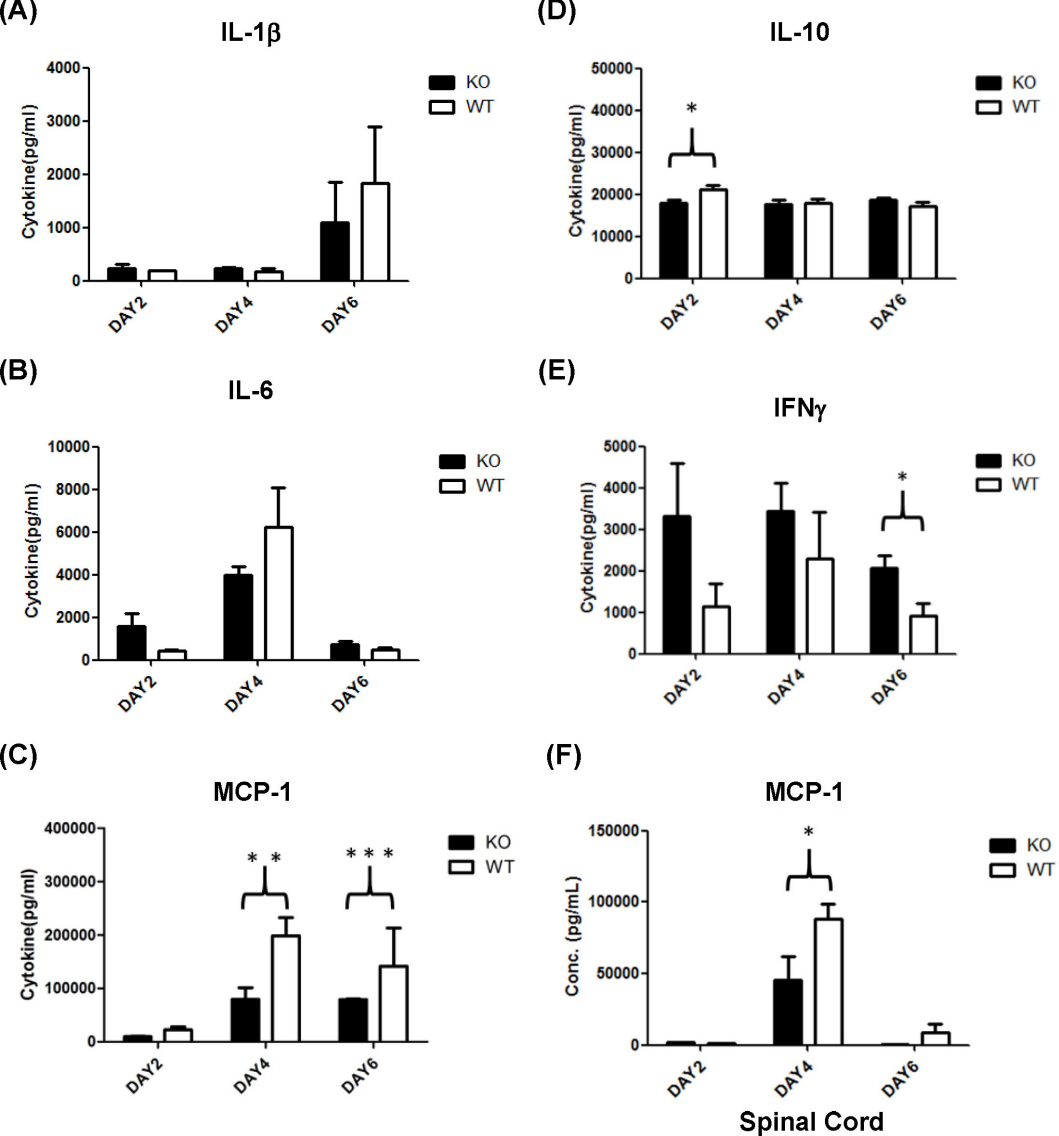
Cytokine expression in the serum of WT and PLG-KO mice. Seven-day-old WT (white blocks) and PLG-KO (black blocks) mice were infected with 5 × 10^4^ pfu/mouse EV-A71 intraperitoneally (*n* = 5). Mice were sacrificed at 2, 4, and 6 days post-infection, and serum/spinal cord samples were collected. The expression of cytokines was evaluated. Cytokine concentrations were presented as pg/mL of the specimen. (**A**) IL-1β, (**B**) IL-6, (**C**) MCP-1, (**D**) IL-10, (**E**) IFN-γ, and (**F**) MCP-1 in the spinal cord. The nonparametric Mann-Whitney *U*-test determined each group’s statistical significance, the mean ± SD. *P < 0.05, **P < 0.01, and ***P < 0.001.

In severe cases of EV-A71 infection, an intense immune response, particularly in the form of a cytokine storm, may induce inflammation or meningitis. Thus, we evaluated serum cytokine levels, such as IL-1β*,* IL-6, IL-10, MCP-1, and IFN-γ in EV-A71 infected PLG-KO and WT mice at 2, 4, and 6 days post-infection. The expression of serum IL-1β was higher on day 6 post-infection than on days 2 and 4 in both groups of mice, though the differences were not statistically significant (Fig. 5A). The levels of serum IL-6 were higher on day 4 than on days 2 and 6 post-infection in both groups of mice (Fig. 5B). Still, there was no statistical significance between groups of mice. It should be noted that the levels of serum MCP-1 were significantly higher in EV-71-infected WT mice than in PLG-KO mice at 4 and 6 days post-infection (Fig. 5C). Serum IL-10 level was significantly higher in EV-71-infected WT mice than in PLG-KO mice at 2 days post-infection (Fig. 5D). Still, it showed no significant differences at 4 and 6 days post-infection between the two groups of mice after EV-A71 infection (Fig. 5D). The serum level of IFN-γ was significantly lower in WT mice than in PLG-KO mice at 6 days post-infection (Fig. 5E). However, the levels of IFN-γ showed no differences at 2 and 4 days post-infection between the two groups of mice after EV-A71 infection (Fig. 5E). In addition to serum, we also determined the MCP-1 expression in the spinal cord of EV-A71-infected WT and PLG-KO mice. Similar to the observation in serum, the spinal cord MCP-1 expression was significantly higher in EV-71-infected WT mice than in PLG-KO mice at 4 days post-infection (Fig. 5F).

## DISCUSSION

The attachment of the virus to the cell surface receptor is a critical step for viral entry and infection. The ability to interact with specific receptors determines the host range, tissue tropism, and pathogenesis (36). SCARB2 and PSGL-1 are known EV-A71 receptors. However, EV-A71 can still infect host cells after blocking SCARB2 or PSGL-1 with monoclonal antibodies or knocking down the expression of these receptors (16, 18). Therefore, unknown receptors or attachment factors for EV-A71 may exist. SCARB2 and PSGL-1 are highly glycosylated proteins. Several studies have also demonstrated that carbohydrates or glycoproteins could mediate EV-A71 infection, such as sialylated glycans, heparan sulfate, and NCL (19, 21, 23, 37). Our previous studies applied a glycoproteomic approach to identify several EV-A71-interacting glycoproteins (23). Glycoprotein extraction included RD and SK-N-SH cell membrane protein isolation, lectin affinity column elution, neuraminidase treatment to diminish the interference of sialic acid, immunoprecipitation with EV-A71, and protein identification using LC/MS/MS. PLG was one of the identified EV-A71 interacting glycoproteins.

Several studies on PLGs involved in viral infections have been reported. Dengue virus infection can induce PLG cross-reactive autoantibodies that may enhance PLG activation and contribute to hemorrhage in patients with dengue hemorrhagic fever or shock syndrome (38). For influenza viruses, proteolytic cleavage of hemagglutinin (HA) is essential for infection. The virions can activate PLG and subsequently permit HA cleavage through the host cellular protein ANXA2 incorporated into influenza A virus particles (33). Infection with influenza A virus also increases PLG binding to the surface of infected cells. Riteau and colleagues used an animal model to demonstrate that PLG could induce lung inflammation upon influenza A virus infection. This contributes to the pathogenesis of IAV through fibrinolysis activation (39). Other viruses also exploit ANXA2 to increase their replication, including cytomegalovirus (40), rabbit vesivirus (41), and human immunodeficiency virus (42).

PLG-deficient (Plg^−/−^) mice have been widely studied to understand the role of PLG in various physiological and pathological processes, including inflammation and blood clotting. These mice lack the precursor of plasmin and exhibit a profound defect in fibrinolysis, leading to the accumulation of fibrin deposits in tissues and blood vessels (43). This impaired fibrin clearance can exacerbate inflammation by acting as a scaffold for immune cell recruitment and activation (44). These mice show delayed wound healing due to a lack of plasmin-mediated extracellular matrix remodeling. The absence of plasmin hampers the migration of inflammatory and reparative cells to the wound site, prolonging the inflammatory phase of healing (45, 46). The accumulation of fibrin promotes prolonged activation of other immune cells, leading to tissue damage. PLG deficiency may lead to reduced survival rates during infection but may confer protective effects during sepsis by modulating inflammatory cytokine production (47). PLG also plays multifaceted roles in a cytokine storm, an excessive, dysregulated immune response characterized by the overproduction of pro-inflammatory cytokines (48, 49). Plasmin, the active form of PLG, can cleave and activate matrix metalloproteinases (MMPs). MMPs can modulate the inflammatory microenvironment by breaking down matrix proteins and releasing cytokines and chemokines (47). These cytokines can exacerbate the inflammatory cascade, fueling the cytokine storm. These interactions between PLG and several cell types, such as endothelial cells, macrophages, and neutrophils, trigger the release of pro-inflammatory mediators, including IL-6, TNF-α, and IFN-γ, the hallmark cytokines of a cytokine storm (50).

The current study found that the knockdown of PLG decreased EV-A71 binding to RD cells (Fig. 1C), and adding PLG promoted EV-A71 binding to RD cells (Fig. 1B). The PLG-EV-A71 interaction (Fig. 1D through F) suggested that serum PLG may bind with EV-A71 in the bloodstream, followed by interacting with cell surface PLG receptors and facilitating virus infection. PLG receptors such as ANXA2 may also be involved in EV-A71 attachment (20). However, PLG interacted with NCL, not ANXA2, during EV-A71 infection (Fig. S7). In addition, the binding of PLG to cell surface receptors may facilitate EV-A71 attachment and activate PLG to form plasmin, which participates in EV-A71-infection-induced cytokine storms.

The mechanisms of PLG-mediated EV-A71 pathogenesis remain unclear. PLG can cleave HA to assist IAV replication via ANXA2, an EV-A71 attachment factor (32, 33). In addition, PLG contributes to the pathogenesis of IAV by activating fibrinolysis. PLG-KO mice are more resistant to IAV-induced death, and cytokine levels in bronchoalveolar lavage of PLG-KO mice are lower than those in WT mice (39). Therefore, PLG-mediated fibrinolysis increases vascular permeability, allowing the recruitment of inflammatory cells to the site of infection (39). Our study observed lower clinical scores, mortality, and serum cytokine expression in EV-A71-infected PLG-KO mice (Fig. 2 and 5), suggesting that PLG may participate in EV-A71-induced immune responses and cytokine storms. The detailed mechanisms by which PLG increases vascular permeability through fibrinolysis activation or the involvement of PLG in the immune system during EV-A71 infection should be further investigated. Further, the role of plasmin in EV-A71 infection was not evaluated in the present study.

This study found that PLG-KO mice are more resistant to EV-A71-induced clinical symptoms and have higher survival rates at 10 days post-infection. The viral load of PLG-KO mice in the spinal cord was lower than that of WT mice at 6 days post-infection (Fig. 2D). Combined with our findings, which indicated that anti-PLG antibodies reduced the binding of EV-A71 to the cell surface (Fig. 1A), we propose that PLG is involved in EV-A71 infection and plays a beneficial role in EV-A71 binding as an attachment factor.

Proinflammatory cytokines such as IL-6, TNF-α, and IL-1β were found to be associated with brainstem encephalitis complicated by pulmonary edema in EV-A71 infections. These pro-inflammatory cytokines are responsible for initiating the inflammatory response during the early phase of disease onset. Anti-inflammatory cytokines such as IL-4, IL-10, and IL-13 are a series of immunoregulatory molecules that mediate the pro-inflammatory cytokine response. These cytokines have been reported to be involved in B-cell proliferation, cytokine production inhibition, and cellular immunity suppression. Other cytokines, such as MCP-1 and IP-10, act as chemokines or chemoattractants that guide cell migration. These chemokines are released from various cells in response to bacterial or viral infection. Chemokines can activate cells to initiate an immune response, induce tissue damage, or promote wound healing. Therefore, dysregulation of these chemokines may induce intense inflammatory responses named cytokine storm.

In this study, IL-10 and MCP-1 expression were significantly higher in EV-A71-infected WT mice than in PLG-KO mice (Fig. 5C, D and F). PLG may stimulate the production of MCP-1 and cause inflammation of cranial nerves, thus promoting meningitis. PLG was found to recruit monocytes through protease-activated receptor-1, MEK/ERK, and MCP-1 signaling. This may imply that EV-A71 utilizes PLG to stimulate the MEK/ERK1/2 signaling pathway and promote MCP-1 production (51). The recruitment of monocytes by MCP-1 may initiate neuroinflammatory conditions and promote EV-A71-associated meningitis. Based on our findings, we suggest that PLG may be the cause of meningitis in EV-A71 infections. In addition, the active form of PLG, plasmin, can stimulate the expression of cytokines (TNF-α, IL-6, IL-1α/β, and CD40), chemokines (MCP-1/CCL2), tissue factors, and the release of lipid mediators and chemotaxis in purified monocytes (52). Based on these findings, we speculated that PLG may mediate immune cytokines, such as MCP-1, to induce intense inflammation (cytokine storm) during EV-A71 infection. This finding may emphasize the importance of PLG in forming cytokine storms induced by EV-A71 infections.

EV-A71 is a highly pathogenic virus characterized by multiple receptors and attachment factors. PLG-KO mice were used to evaluate the infectivity of EV-A71. We observed that PLG-KO mice expressed fewer clinical symptoms than WT mice and that the viral loads in the PLG-KO mice in some organs were also lower than those in the WT mice at 3 and 6 days post-infection. In the tissue section used to examine meningitis, neural tissues such as spinal cords and brain stem showed a higher EV-A71 signal. The tissues were also infiltrated by significant numbers of immune cells in the case of the WT mice. To determine whether the immune system is affected by PLG and induces meningitis symptoms, we investigated the expression levels of EV-A71-associated cytokines such as IL-1β, IL-6, MCP-1, IL-10, and IFN-γ in serum after EV-A71 infection. Serum IL-10 and MCP-1 expression levels were lower in PLG-KO mice than in WT mice. In contrast, the expression of serum IFN-γ was significantly higher in PLG-KO mice. Comparing the expression levels of MCP-1 in the serum and spinal cords with their clinical symptoms, MCP-1 may be an essential chemokine inducing inflammation and chemotactic leukocytes. These findings suggest that PLG could be used as a target to develop novel drugs or treatments to prevent EV-A71 infection.

## Data Availability

The authors confirm that the data supporting the findings of this study are available in the article and its supplemental material.

## SUPPLEMENTAL MATERIAL

Supplemental table and figures spectrum.03311-24-s0001.docx Table S1 and Fig. S1 to S7.

ASM does not own the copyrights to Supplemental Material that may be linked to, or accessed through, an article. The authors have granted ASM a non-exclusive, world-wide license to publish the Supplemental Material files. Please contact the corresponding author directly for reuse.
