# Supplementary material for: Plasminogen deficiency reduces disease severity and immune responses in enterovirus A71-infected mice
Source: Microbiol Spectr. 2025 May 16;13(7):e03311-24. doi: 10.1128/spectrum.03311-24 (PMC12210968; doi:10.1128/spectrum.03311-24)
Supplement: Supplemental table and figures — Table S1 and Fig. S1 to S7. [file spectrum.03311-24-s0001.docx]

**Supplementary Table 1: PCR primers, products, and conditions.**

| Primer | | | | | | | | | | | | |
| --- | --- | --- | --- | --- | --- | --- | --- | --- | --- | --- | --- | --- |
| Primer name | | | | | Sequence | | | | | | | |
| 1 | PLG-486 | | | | 5′-TGTGGGCTCTAAAGATGGAACTCC-3′ | | | | | | | |
| 2 | PLG-487 | | | | 5′-GTGCGAGGCCAGAGGCCACTTGTGTAGCG-3′ | | | | | | | |
| 3 | PLG-488 | | | | 5′-GACAAGGGGACTCGCTGGATGGCTA-3′ | | | | | | | |
| Reaction conditions | | | | | | | | | | | | |
| Component | | | Concentration | | | Volume | | Step | Temperature, ℃ | | Time, sec | Number of cycles |
| H_2_O | | |  | | | 10.325 | μL | 1 | 95 | | 2 min | 1 |
| PCR buffer | | | 10x | | | 1.5 | μL | 2 | 95 | | 35 sec | 45 |
| dNTP | | | 10 mM | | | 1.2 | μL | 3 | 60 | | 35 sec |  |
| Primer 1 | | | 20 μM(ρmol/μL) | | | 0.3 | μL | 4 | 72 | | 50 sec |  |
| Primer 2 | | | 20 μM(ρmol/μL) | | | 0.3 | μL | 5 | 72 | | 10 min | 1 |
| gDNA | | | 50~250 ng/μL | | | 1 | μL |  |  | |  |  |
| Taq | | | 5 U/μL | | | 0.075 | μL |  |  | |  |  |
| Total | | | | | | 15 | μL |  |  | |  |  |
| PCR product | | | | | | | | | | | | |
|  | | Primer set | | | | | | Product size | |  | | |
|  |  | 5′primer | | 3′primer | | | |  |  |  |  |  |
| 1 | | PLG-487 | | PLG-486 | | | | 190 bp | |  |  |  |
| 2 | | PLG-488 | | PLG-486 | | | | 268 bp | |  |  |  |

**Supplementary Figures**


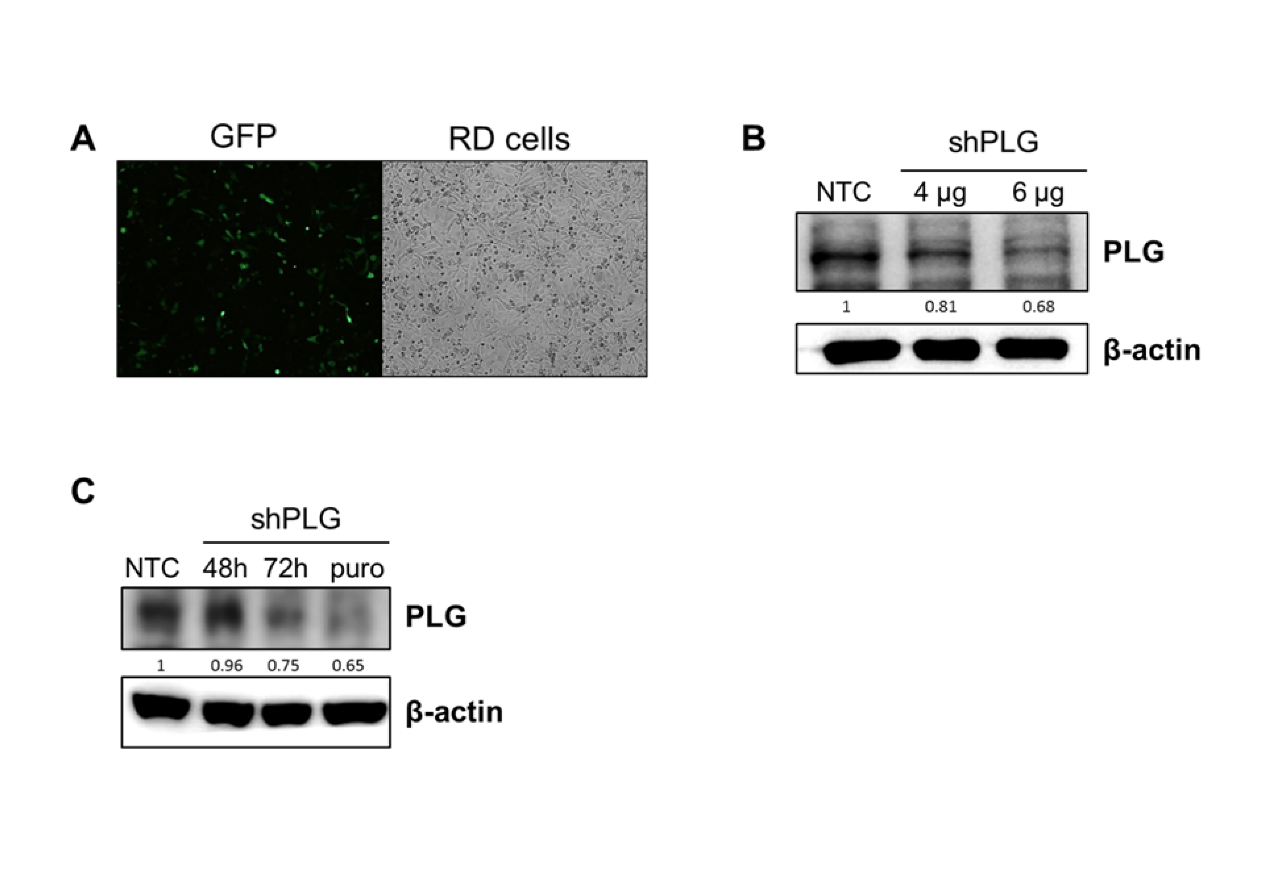


**Supplementary Figure 1**: (A) The 4 μg of GFP-based plasmids were transfected into RD cells for 48 hours and were observed using a fluorescence microscope. (B) The 0, 4, and 6 μg of PLG shRNA plasmids were transfected into RD cells for 48 hours. The protein expression was detected by Western blotting using anti-PLG (1:1000) for primary antibody and anti-mouse IgM (1:10000) for secondary antibody. (C) The RD cells (2 x 10^5^ cells/well) were seeded in a 6-well plate and were infected with the lentivirus (M.O.I=3) for 48 and 72 hours, or added 2 μg/ml of puromycin in DMEM after 24 hours of lentivirus infection. The protein expression was detected by Western blotting and quantified with Image J. NTC: negative control.

1. (B)

**
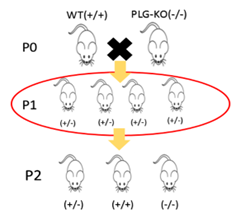
**
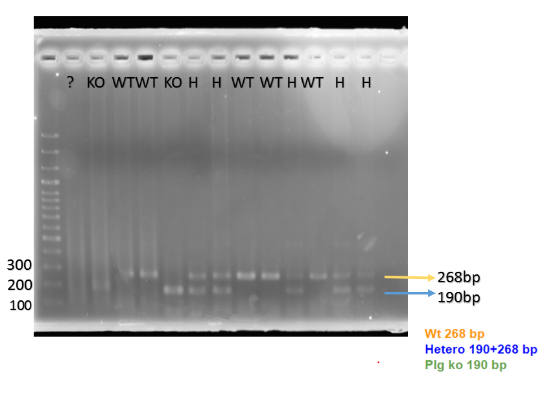


**Supplementary Figure 2**: **Establishing of PLG-KO mice (heterozygous).** (A) PLG-deficient mice (homozygous) were provided by Dr. Hua-Lin Wu, who purchased them from The Jackson Laboratory (Bar Harbor, ME, USA). We obtained PLG-KO (P1, heterozygous) mice by mating PLG-deficient mice with WT mice. We mated PLG-KO mice with PLG-KO mice (P1 x P1) to obtain WT, PLG-KO mice (heterozygous), and PLG-deficient mice (homozygous) at the same birth. P2 mice were used for EV-A71 infection throughout the experiments. (B) 7-day-old wild-type (WT) and PLG-KO mice were genotyped by polymerase chain reaction (PCR). WT mice express a single band on 268bp, and KO mice express a single band on 190bp, and hetero mice will express two bands on 190bp and 268bp.


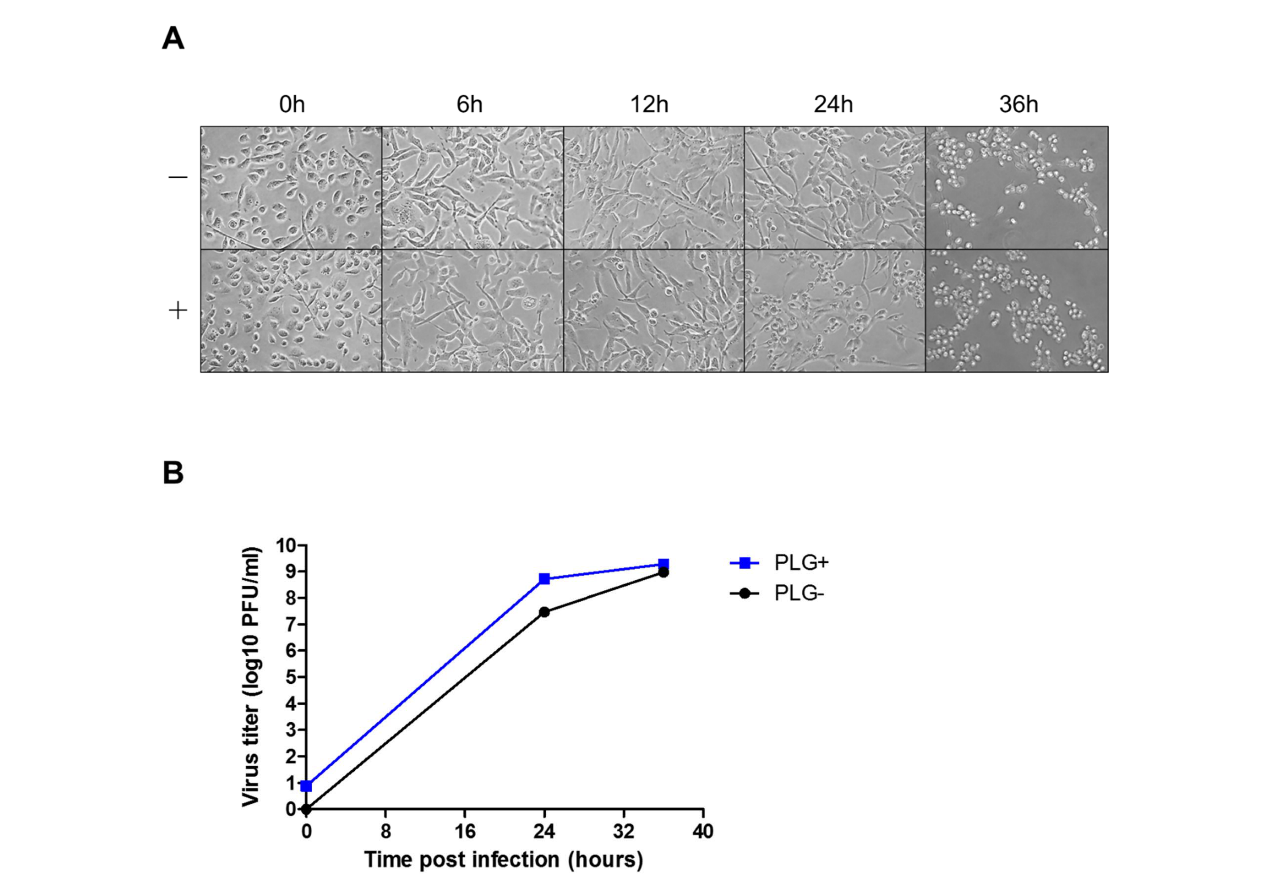


**Supplementary Figure 3**: The RD cells (1.5 x 10^5^ cells/well) were seeded in a 12-well plate. Purified EV71 6356 (M.O.I = 0.1) were incubated with or without 200 μg/mL of PLG in serum-free DMEM for 1 hour at 37^o^C. After incubation, the EV71 were infected to RD cells for 1 hour at 35^o^C. The unbound virus was removed with 1 mL of PBS and incubated in serum-free DMEM. (A) The cytopathic effects were observed by light microscopy. (－, without PLG, ＋, with PLG) (B) The viral supernatants were collected, and the virus titers were determined by plaque assay.





**Supplementary Figure 4**: The bindings of EV-A71 (positive control), coxsackievirus A10 (CA10), and A16 (CA16) with PLG were assessed using a EnSpire system.

**
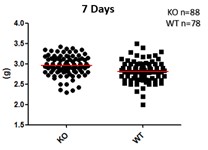

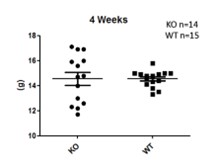

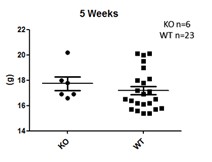
**

**Supplementary Figure 5.** The body weight of WT and PLG-KO mice (heterozygous) was evaluated at 7 days, 4 weeks, and 5 weeks of birth. PLG-KO mice showed normal body weight and appearance, like wild-type mice.


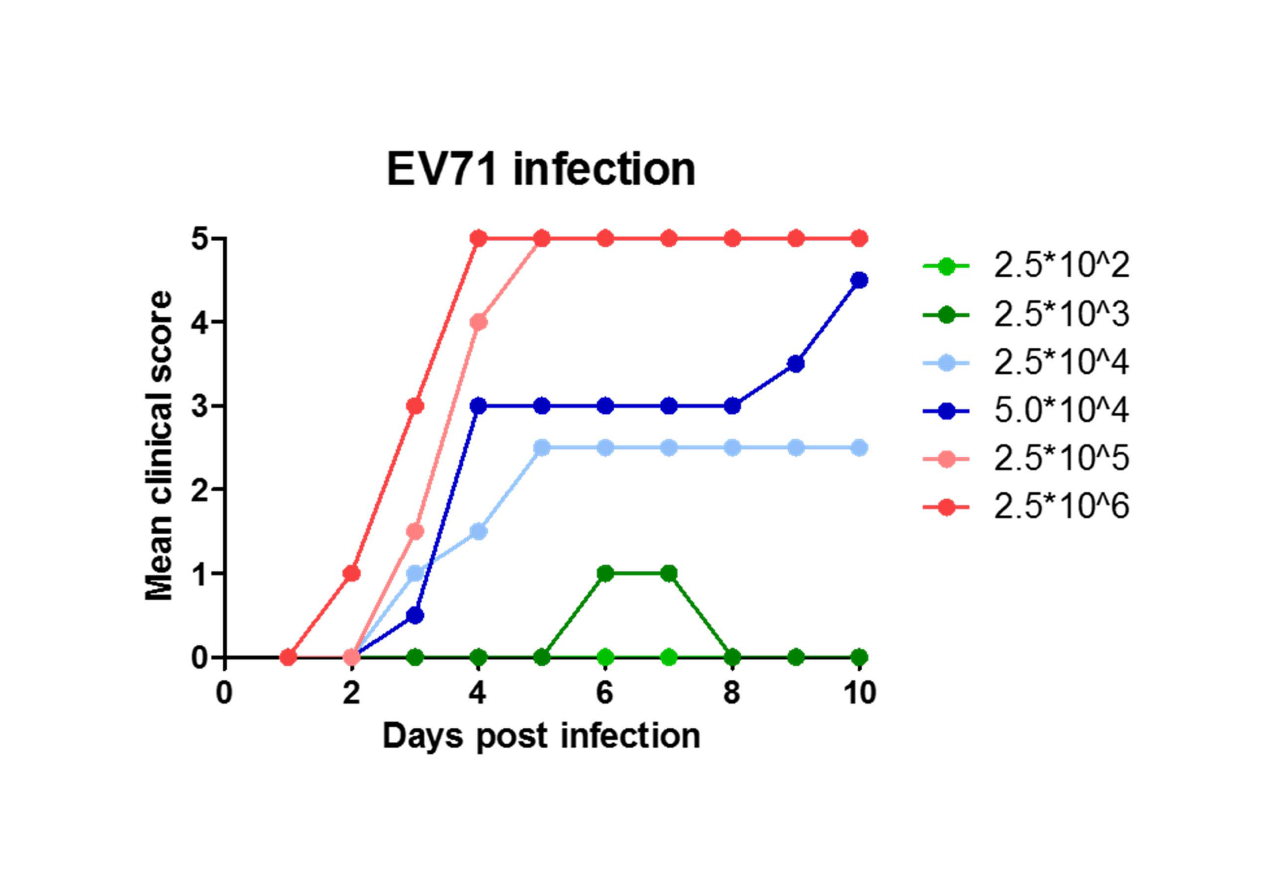


**Supplementary Figure 6**: The WT mice were infected with 2.5 x 10^2^ to 2.5 x 10^6^ pfu/mouse of EV71 MP4 strain (n=3 for each dose). The clinical scores were recorded from day 0 to day 10. Clinical scores: 0, healthy; 1, reduced motility; 2, limb weakness; 3, limb paralysis; 4, moribund; 5, death.


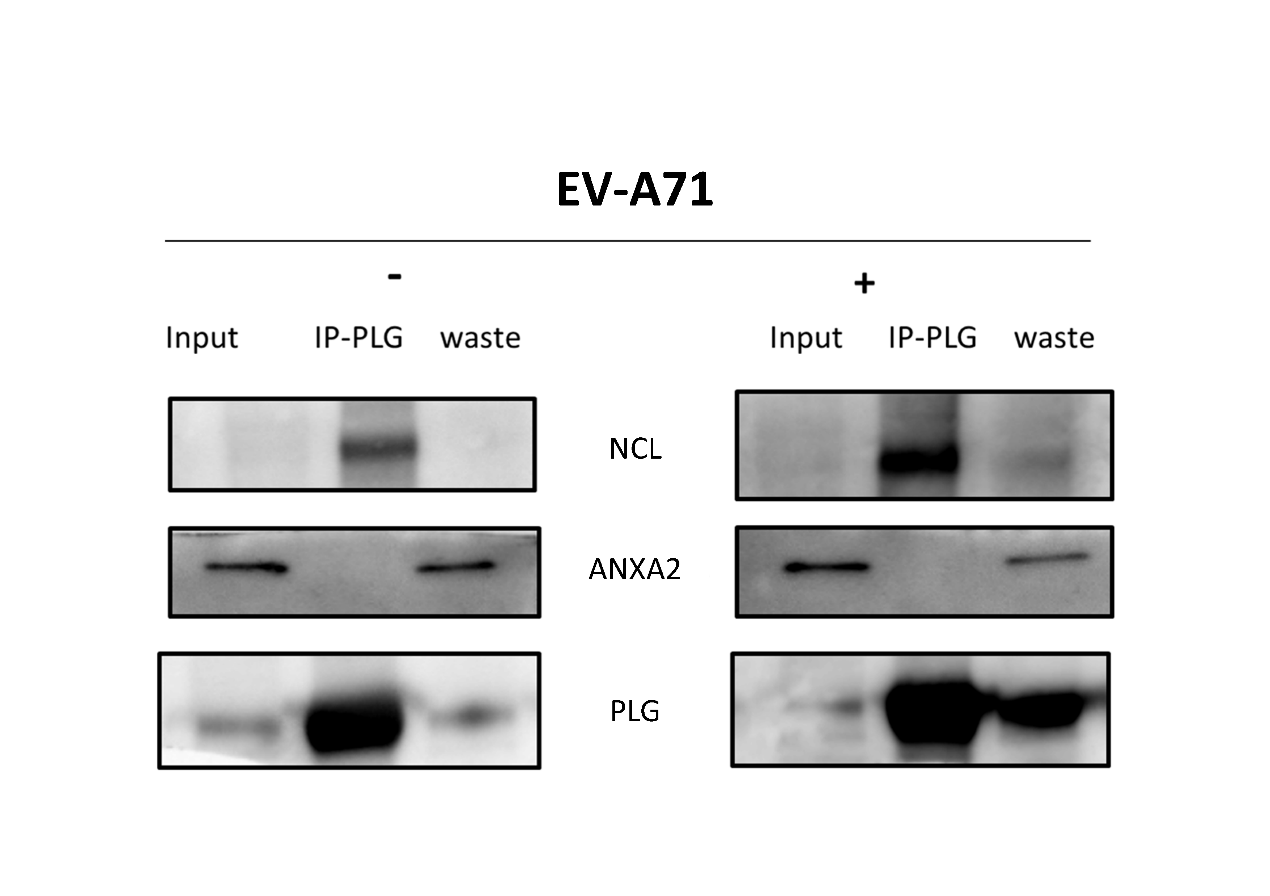


**Supplementary Figure 7**: **Plasminogen interacted with nucleolin but not annexin A2 during EV-A71 infection.** The RD cells were infected with or without a 100× CCID_50_ dose of EV-A71 at 35^o^C for 1 hr. The lysates were harvested and precipitated with anti-PLG antibody. The plasminogen, nucleolin, and annexin A2 were detected by Western blotting.
